# The Effect of Low-Dose Marine *n*-3 Fatty Acids on Plasma Levels of sCD36 in Overweight Subjects: A Randomized, Double-Blind, Placebo-Controlled Trial

**DOI:** 10.3390/md11093324

**Published:** 2013-08-30

**Authors:** Stine Krogh Venø, Michael René Skjelbo Nielsen, Søren Lundbye-Christensen, Erik Berg Schmidt, Aase Handberg

**Affiliations:** 1 Department of Cardiology, Center for Cardiovascular Research, Aalborg University Hospital, Aalborg 9000, Denmark; E-Mails: mrsn@rn.dk (M.R.S.N.); solc@rn.dk (S.L.-C.); ebs@rn.dk (E.B.S.); 2 Department of Clinical Biochemistry, Aalborg University Hospital, Aalborg 9000, Denmark; E-Mail: aaha@rn.dk

**Keywords:** *n*-3 PUFA, CD36, adipose tissue, cardiovascular disease, randomised controlled trial

## Abstract

CD36 is a scavenger receptor involved in lipid uptake and inflammation. Recently, non-cell-bound CD36 (sCD36) was identified in plasma and suggested to be a marker of lipid accumulation in the vessel wall. Marine *n*-3 polyunsaturated fatty acids (PUFA) may have cardioprotective effects. This study evaluated the effect of marine *n*-3 PUFA on sCD36 levels in overweight subjects. Fifty overweight subjects were randomized to 1.1 g of *n*-3 PUFA or 2 g of olive oil daily for six weeks. Neutrophils were isolated at baseline and after six weeks of treatment while an adipose tissue biopsy was obtained at baseline. The content of *n*-3 PUFA in adipose tissue and neutrophils was analyzed by gas chromatography, while plasma levels of sCD36 were determined using an enzyme-linked immunosorbent assay (ELISA). After six weeks of supplement plasma sCD36 did not differ between supplements (*P* = 0.18). There was no significant correlation between plasma sCD36 levels and *n*-3 PUFA in neutrophils at baseline (*r* = −0.02, *P* = 0.88), after six weeks supplement (*r* = −0.03, *P* = 0.85) or in adipose tissue (*r* = 0.14, *P* = 0.34). This study therefore does not provide evidence for a cardioprotective effect of *n*-3 PUFA acting through a CD36-dependent mechanism.

## 1. Introduction

Epidemiological studies have shown an inverse association between fish consumption and morbidity or mortality from cardiovascular disease (CVD) [1,2,3,4]. This beneficial effect has been attributed to the content of long-chained *n*-3 polyunsaturated fatty acids (PUFA) in seafood although other components of fish e.g. selenium, vitamins and peptides may also contribute [1,3,4,5,6]. Data from *in vitro* experiments and animal studies have supported the hypothesis of a beneficial effect of marine *n*-3 PUFA on CVD, and several (but not all) randomized controlled clinical trials [7,8,9,10] have shown a preventive effect of *n*-3 PUFA on CVD. Several mechanisms including antithrombotic, antiarrhythmic, triglyceride lowering and antihypertensive effects of marine *n*-3 PUFA have been suggested as explanation for these effects. Recently, much focus has been on anti-inflammatory effects of *n*-3 PUFA [11] as inflammation is considered of major importance for the development and progression of atherosclerosis and its clinical complications [12]. 

CD36 is a membrane protein containing two transmembrane areas, two short intracellular domains and a large extra-cellular loop [13]. In relation to atherosclerosis, CD36 in macrophage membranes serves as a scavenger receptor for oxidized low-density lipoprotein [14]. Binding of oxidized low density lipoprotein (oxLDL) to CD36 receptors leads to accumulation of cholesterol in the macrophages thereby initiating foam cell formation. OxLDL also interacts with CD36 on monocytes making them pro-inflammatory, and platelets leading to activation and an increased risk of cardiovascular events. Recently, a non-cell bound form of CD36 (sCD36) has been discovered in plasma [15], and sCD36 is believed to reflect tissue levels of CD36 and has been suggested to be a marker of lipid accumulation in the vessel wall [16,17].

Obesity is associated with an increased risk of CVD, at least partly related to an associated increased occurrence of risk factors for CVD like diabetes mellitus, hypertension and dyslipidaemia, but probably also low-grade chronic inflammation [18,19]. 

Little is known about the association between *n*-3 PUFA intake and levels of CD36. Studies have been performed in LDL receptor null mice and human dermal microvascular endothelial cells but not in humans [20,21].

The aim of the present study therefore was to investigate whether a supplement with marine *n*-3 PUFA in a low—but generally recommended dose for prevention of CVD—of approximately 1 g per day decreased plasma levels of sCD36 in overweight subjects. Secondary aims were to assess whether short-term and long-term biomarkers of *n*-3 PUFA intake were correlated to sCD36.

## 2. Results and Discussion

### 2.1. Subject Characteristics

Fifty overweight subjects were included in the study, but one subject, randomized to olive oil, dropped out due to gastrointestinal discomfort. No changes in medications or diets in either group during the 6-week period of supplementation were reported. 

There were no significant differences in baseline characteristics between the two treatment groups except that more subjects were treated with acetylsalicylic acid (ASA) and β-blockers in the *n*-3 PUFA group (Table 1). The subjects’ mean age was 57 years, mean BMI was 30.2 kg/m^2^ and mean waist circumference was 107.7 cm for men and 97.2 cm for women. 

At baseline, there were no significant differences in the fatty acid composition in neutrophils between the two groups. After six weeks of supplementation, the neutrophil content of marine *n*-3 PUFA (eicosapentaonoic acid (EPA), docosahexaenoic acid (DHA) and docosapentaenoic acid (DPA)) increased significantly in the *n*-3 PUFA group when compared to controls (*P* < 0.01). There was no alteration in the content of saturated fatty acids neither within nor between the *n*-3 group and the control group (Table 2). These results have previously been published [22].

**Table 1:** Baseline characteristics.

		Control (*n* = 25)		*n*-3 PUFA (*n* = 25)	
Gender					
	Male	12 (48%)		12 (48%)	
	Female	13 (52%)		13 (52%)	
Age (years)		55.4 ± 9.5		58.0 ± 7.4	
BMI (kg/m^2^)		29.5 ± 3.3		30.8 ± 4.2	
Waist circumference (cm)					
	Male	106.4 ± 7.7		107.7 ± 7.7	
	Female	94.6 ± 6.9		99.8 ± 12.8	
Smoking					
	Smokers	3 (12%)		5 (20%)	
	Non-smokers	22 (88%)		20 (80%)	
Medication					
ASA		3 (12%)		15 (60%)	
Lipid-lowering drugs		19 (68%)		23 (76%)	
Insulin		0 (0%)		1 (4%)	
Oral anti-diabetic drugs		0 (0%)		3 (12%)	
β-blockers		3 (12%)		13 (52%)	
Other anti-hypertensive drugs		19 (48%)		17 (44%)	
Disease					
Dyslipidemia		17 (68%)		19 (76%)	
Hypertension		13 (52%)		10 (40%)	
DM2		0 (0%)		4 (16%)	
AMI		1 (4%)		3 (12%)	

Numerical variables are given as means ± SD and categorical variables as numbers and percentages; Abbreviations: BMI, Body mass index; ASA, Acetylsalicylic acid; DM2, Diabetes mellitus type 2; AMI, Acute myocardial infarction.

**Table 2:** Fatty acid profiles at baseline and after supplements and changes with supplements.

	Baseline							6 weeks							Change: Baseline—6 weeks						
	Control			*n*-3 PUFA			*P* ^a^	Control			*n*-3 PUFA			*P* ^b^	Control			*n*-3 PUFA			*P ^c^*
Neutrophils (Wt%)	Mean		SD	Mean		SD		Mean		SD	Mean		SD		Mean		SD	Mean		SD	
14:0	0.17	±	0.04	0.16	±	0.03	0.28	0.15	±	0.05	0.16	±	0.04	0.41	−0.02	±	0.05	0.00	±	0.03	0.12
16:0	12.04	±	0.60	11.81	±	0.59	0.18	11.91	±	0.62	11.83	±	0.67	0.69	−0.10	±	0.47	0.03	±	0.42	0.32
18:0	17.20	±	0.59	16.98	±	0.49	0.15	17.16	±	0.55	17.00	±	0.54	0.30	−0.03	±	0.50	0.02	±	0.28	0.65
18:1*n*-9	31.53	±	1.59	31.86	±	1.69	0.41	31.76	±	1.49	32.46	±	1.56	0.47	0.37	±	1.74	0.60	±	1.07	0.58
18:2*n*-6 LA	9.03	±	0.93	8.64	±	0.67	0.10	8.77	±	0.73	8.88	±	0.94	0.63	−0.27	±	0.87	0.25	±	0.93	0.05
20:4*n*-6 AA	13.27	±	1.11	13.36	±	0.85	0.75	13.43	±	1.10	12.17	±	0.93	<0.01	0.10	±	0.94	−1.20	±	0.73	<0.01
20:5*n*-3 EPA	0.58	±	0.30	0.68	±	0.33	0.25	0.57	±	0.27	1.36	±	0.35	<0.01	0.04	±	0.18	0.68	±	0.31	<0.01
22:5*n*-3 DPA	1.62	±	0.46	1.74	±	0.44	0.34	1.61	±	0.30	2.39	±	0.79	<0.01	−0.03	±	0.35	0.64	±	0.53	<0.01
22:6*n*-3 DHA	1.34	±	0.40	1.40	±	0.51	0.61	1.33	±	0.31	1.65	±	0.58	<0.01	−0.00	±	0.22	0.25	±	0.34	<0.01
Adipose tissue (Wt%)																					
14:0	2.06	±	0.38	2.04	±	0.35	0.86														
16:0	19.63	±	1.91	19.96	±	1.95	0.55														
18:0	2.75	±	0.61	2.78	±	0.67	0.89														
18:1*n*-9	48.10	±	1.63	47.96	±	1.63	0.76														
18:2*n*-6 LA	10.36	±	1.08	10.25	±	0.80	0.66														
20:4*n*-6 AA	0.46	±	0.11	0.52	±	0.13	0.09														
20:5*n*-3 EPA	0.11	±	0.06	0.12	±	0.05	0.80														
22:5*n*-3 DPA	0.35	±	0.12	0.35	±	0.10	0.98														
22:6*n*-3 DHA	0.29	±	0.14	0.27	±	0.15	0.68														

Fatty acid profiles are shown at baseline, after a daily supplement with 1.1 g of marine *n*-3 fatty acids and after control oil (2.0 g of olive oil); Results are given as means ± SD (% of total fatty acids), unadjusted analysis; ^a ^*P*-values test the hypothesis of difference between the treatment groups at baseline; ^b ^*P*-values test the hypothesis of difference between treatment groups after 6 weeks of supplementation; ^c^* P*-values test the hypothesis of difference between treatment groups in changes from baseline to 6 weeks.

**Figure 1:**
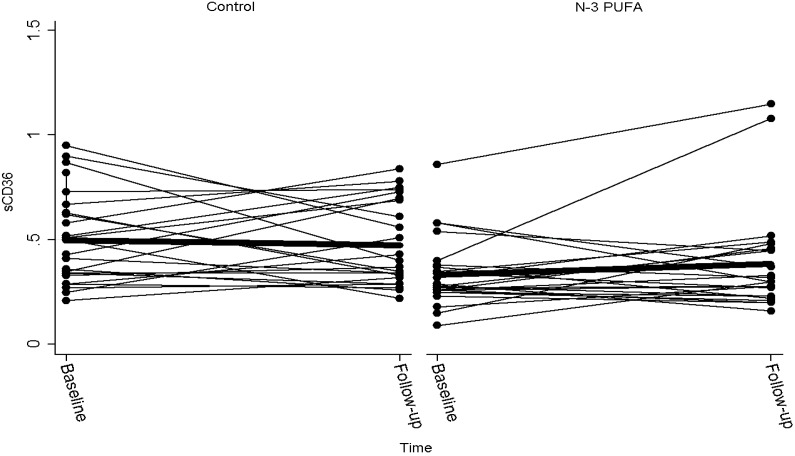
Plasma sCD36 before and after six weeks of supplementation with control (olive oil) or marine *n*-3 PUFA. One subject with sCD36 of 0.4 at baseline and 3.12 after intake of *n*-3 PUFA was considered an outlier and removed from the figure. The bold line indicates mean value.

At baseline, sCD36 levels were significantly higher in the control group than in the *n*-3 PUFA group (*P* < 0.01). Despite a small increase in sCD36 in the *n*-3 PUFA group and a small decrease in the control group, there was no significant difference between treatments after 6 weeks (*P* = 0.18) (Table 3 and Figure 1). The mean dietary intake of marine *n*-3 fatty acid was 0.5 g/day with no difference between groups. Also, there was no significant association between intake of either fatty or lean fish and sCD36 (*P* = 0.98).

**Table 3:** sCD36 at baseline and day 42.

	sCD36 baseline	sCD36 day 42	Change in CD36	*P*
Controls	0.50 (0.41, 0.59)	0.47 (0.39, 0.56)	−0.01 (−0.11, 0.09)	
*n*-3 PUFA	0.34 (0.27, 0.40)	0.49 (0.25, 0.74)	0.16 (−0.08, 0.39)	
Difference	0.16 (0.05, 0.27)	−0.02 (−0.28, 0.24)	−0.17 (−0.42, 0.08)	0.18

Results are given as means with 95% confidence interval.

Plasma levels of sCD36 showed no significant association with the content of EPA (*r* = 0.01, *P* = 0.92), DPA (*r* = 0.18, *P* = 0.20) and DHA (*r* = 0.12, *P* = 0.40) or total *n*-3 PUFA (*r* = 0.14, *P* = 0.34) in adipose tissue at baseline, and this result was not affected by adjustment for possible confounding effects of age, gender and BMI.

Changes in sCD36 from baseline to day 42 showed no correlation with changes in *n*-3 PUFA in neutrophils (*r* = 0.07, *P* = 0.64), and there was no significant association between sCD36 and *n*-3 PUFA neither at baseline (*r* = −0.02, *P* = 0.88) nor after 6 weeks’ supplement (*r* = −0.03, *P* = 0.85). After adjusting for confounding effects of age, gender and BMI, the results remained insignificant.

### 2.2. Discussion

Several studies have indicated that sCD36 may reflect tissue CD36 levels, and sCD36 has been proposed to be a marker of CVD. Supplementation with marine *n*-3 PUFA is associated with cardiovascular protection, and we hypothesized a reduction in CD36 after supplementation with marine *n*-3 PUFA. In the present study, we measured plasma levels of sCD36 at baseline and after six weeks of treatment with *n*-3 PUFA or a control oil (olive oil). We measured *n*-3 PUFA in both neutrophils and adipose tissue. The composition of adipose tissue reflects long-term (months/years) dietary fatty acid intake [23], whereas the composition of neutrophils reflects short-term intake (weeks) of fatty acids. In the present study, we found no effect of supplementing *n*-3 PUFA and no correlation between long or short-term intake of *n*-3 PUFA and plasma levels of sCD36, and therefore the cardioprotective effect of *n*-3 PUFA seems not to be mediated through a modulation of CD36 levels.

The effect of marine *n*-3 PUFA and CD36 has been studied by others. Thus, in an animal study [21] LDL receptor null mice were fed a high saturated fat and cholesterol diet with different PUFA including *n*-3 PUFA for 32 weeks. Membrane-bound CD36 from macrophages was extracted, and there was no significant association between intake of *n*-3 PUFA and CD36, an observation that is in line with our results. Madonna *et al.* [20] studied the effect of *n*-3 PUFA on CD36 using human dermal microvascular endothelial cells. After stimulation with insulin, an increased surface expression of CD36 was observed; this is in line with the consistent finding of elevated sCD36 in conditions with hyperinsulinemia, such as obesity, type 2 diabetes mellitus and polycystic ovary syndrome [17]. Interestingly, EPA and DHA attenuated constitutive and insulin-induced CD36 expression in microvascular endothelial cells in this setup [20]. This effect on cell cultures could not be confirmed in our study in overweight patients. In this complex set-up it may be assumed that plasma sCD36 mirrors tissue CD36 expression. Changes in CD36 originating from microvascular endothelial cells may not be visible when mixed with total sCD36, or the effect seen in cell cultures could have been obtained through an improvement of insulin sensitivity. 

Recently another randomized placebo-controlled study with *n*-3 PUFA and olive oil was published [24]. Thirty patients with coronary artery disease and triglycerides >3 mmol/L were randomized to either 1 g of *n*-3 PUFA/day (465 mg DHA, 375 mg EPA), 2 g of *n*-3 PUFA/day or 1 g of olive oil/day. All subjects were treated with aspirin and statins. After two weeks of treatment, the subjects who received 1 or 2 g of *n*-3 PUFA had a reduction in platelet reactivity, but there was no significant change in surface expression of platelet-CD36. 

In our study we used a low dose of *n*-3 PUFA, and we cannot exclude that the dose was too low to exert any effect on sCD36. However, we used the dose of approximately 1 g of marine *n*-3 PUFA recommended by the European Society of Cardiology and the American Heart Association for prevention of CVD [25,26]. For unknown reasons, sCD36 was significantly lower in the *n*-3 PUFA group than in the control group at baseline which might potentially render it more difficult to show any effect of *n*-3 PUFA. Another important limitation of our study was that 42 of the participants were treated with lipid-lowering drugs and 36 were treated with other anti-hypertensive drugs. Furthermore, randomization was not successful with respect to use of aspirin and β-blockers which was higher in the *n*-3 PUFA group than in controls. Medications may be a confounder to a potential *n*-3 PUFA-induced alteration in CD36 as a previous study reported that statins reduce CD36 [27], and type 2 diabetic patients treated with aspirin had lower levels of sCD36 compared to a non-treated control group [28]. Finally, one subject in the *n*-3 PUFA group had a sCD36 value of 3.12 at day 42 which is extremely high. To make sure it was not an analytical error, the measurement of sCD36 was repeated and confirmed. We chose not to exclude the subject though the sCD36 is more than 6 × SD from the mean sCD36. The study cohort comprised 50 subjects, and it could be argued that a larger cohort might have given significant and clear-cut results. Also, it could be questioned whether olive oil is actually inert or has an effect on CD36. Indeed, a study of eleven healthy subjects who ingested 50 mL olive oil after 1 h showed an upregulation in CD36 in peripheral blood mononuclear cells [29]. Results were borderline significant and 6 h after ingestion of the high olive oil dose CD36 had returned to basal values. Finally, it could be argued that monocytes would have been better to use than neutrophils.

The study design also had some strengths. It was a randomized controlled study with two parallel groups in a relevant group of overweight patients with an increased risk of vascular disease. We measured *n*-3 PUFA from both neutrophils (short-term intake) and from adipose tissue (long-term intake) from which we could further explore the association between dietary intake of *n*-3 PUFA and plasma sCD36. 

## 3. Experimental Section

### 3.1. Study Subjects

Fifty overweight subjects, aged between 30 and 75, were recruited from the patients of the Department of Cardiology and the medical staff at Aalborg Hospital, Denmark (Table 1). The inclusion criteria were a waist circumference >94 cm for men and >80 cm for women according to the WHO definition of abdominal obesity in a European population [30]. Medications should be stable for at least 6 weeks prior to inclusion, and the participants were asked to keep medications unchanged during the study period. Exclusion criteria were: intake of anti-inflammatory drugs except low-dose acetylsalicylic acid, intake of estrogen/progesterone analogues, poorly regulated diabetes mellitus (HbA1c > 8%), any acute or chronic inflammatory condition or severe renal insufficiency (GFR < 30 mL/min). 

### 3.2. Study Design

The study design has been described in detail elsewhere [22]. Briefly, the study was designed as a double blind randomized and placebo-controlled trial. The participants were randomly assigned to two externally identical capsules of oil containing either 1.1 g marine *n*-3 PUFA (640 mg EPA and 480 mg DHA) or 2.0 g of olive oil daily for six weeks. The study was approved by the local ethical committee, and all participants gave informed consent. The trial was registered at Clinicaltrials.gov (NCT00885053).

### 3.3. Blood and Adipose Tissue Sampling

Blood samples were collected at baseline and after six weeks of supplement from a cubital vein after the participants had been fasting for at least 10 h. Blood samples were anticoagulated with K_3_-EDTA and frozen at −80 °C. To eliminate inter-assay variability, samples from each participant were analyzed in the same analytical run for all parameters. Adipose tissue samples were taken at baseline from subcutaneous fat of the buttocks as previously described [31].

### 3.4. Fatty Acid Composition of Neutrophils and Adipose Tissue

The extraction of neutrophils from blood has previously been described in detail [32], and total fatty acids from neutrophils were separated according to the method of Van Kuijk [33]. A 250 µL cell suspension was extracted into 1.0 mL dichloromethane and 1.0 mL methanol containing BTH 10 mg/100 mL. Next, 1.0 mL dichloromethane and 500 µL H_2_O were added to the samples and centrifuged. Adipose tissue was dissolved in 1.0 mL heptane, methylated for 2 min. at 50 °C using 50 µL 2 M potassium hydroxide in methanol and subsequently centrifuged. Fatty acid composition of neutrophils and adipose tissue was analyzed by gas chromatography using a Varian 3900 GC supplied with a CP-8400 auto sampler and a flame ionisation detector (Varian, Middleburg, The Netherlands). To identify the individual fatty acids, commercially available standards (NU-chek-Prep, Inc., Elysian, MN, USA) were used.

### 3.5. Measurement of sCD36

sCD36 was measured by an in-house enzyme-linked immunosorbent assay (ELISA) using plasma anticoagulated with EDTA as previously described [15]. A pool of EDTA plasma was sonicated three times for 5 s on ice, applied in seven dilutions and used to produce a standard concentration curve, and sCD36 concentrations were expressed relative to the plasma pool (arbitrary units). Internal controls were run in quadruplicate on each plate. Inter-run coefficient of variation for this assay is around 16%.

### 3.6. Food Questionnaire

A food questionnaire was used to collect information of the subjects’ daily intake of fish at baseline, distinguishing between fatty and lean fish as previously described [34].

### 3.7. Statistical Analyses

Differences between treatment groups in changes in sCD36 over time were analyzed by unpaired *t*-test, with Welch correction when variances differed between groups. Associations between biomarkers of short-term (neutrophils) and long-term (adipose tissue) dietary intake of *n*-3 PUFA and plasma sCD36 were assessed by Pearson correlations. As an explanatory analysis, we also assessed these associations with a multiple linear regression model in which selected potential confounders (age, gender and BMI) were included. Logistic transformation was applied when appropriate, and model assumptions were subsequently found to be adequate, using relevant graphical plots.

Statistical tests were two-tailed, and *P* < 0.05 was considered significant. Stata version 11.2 (StataCorp. 2009. Stata: Release 11. Statistical Software: College Station, TX, USA. Stata Corp LP) was used.

## 4. Conclusions

Supplementation with 1.1 g of *n*-3 PUFA for six weeks had no effect on plasma levels of CD36. Furthermore, there was no correlation between sCD36 and *n*-3 PUFA content in neutrophils and adipose tissue. The study therefore does not provide evidence for a cardioprotective effect of marine *n*-3 PUFA acting through a CD36-dependent mechanism. Although it cannot be excluded that other doses of marine *n*-3 PUFA could have an effect on plasma sCD36 levels.
